# Evaluation of the In Vitro Capacity of Anti-Human Cytomegalovirus Antibodies to Initiate Antibody-Dependent Cell Cytotoxicity

**DOI:** 10.3390/microorganisms12071355

**Published:** 2024-07-02

**Authors:** Piera d’Angelo, Federica Zavaglio, Elisa Gabanti, Paola Zelini, Chiara Fornara, Stefano Bernuzzi, Arsenio Spinillo, Daniele Lilleri, Fausto Baldanti

**Affiliations:** 1 Microbiology and Virology Unit, Fondazione IRCCS Policlinico San Matteo, 27100 Pavia, Italy; 2 Department of Molecular Medicine, University of Pavia, 27100 Pavia, Italy; 3 M.A.D. Analisi, 27058 Voghera, Italy; 4 Laboratory Medicine Service, Istituti Clinici Scientifici Maugeri IRCCS, 27100 Pavia, Italy; 5 Service of Immunohematology and Transfusion Medicine, Fondazione IRCCS Policlinico San Matteo, 27100 Pavia, Italy; 6 Department of Clinical, Surgical, Diagnostics and Pediatric Sciences, University of Pavia, 27100 Pavia, Italy; 7 Department of Obstetrics and Gynecology, IRCCS Policlinico San Matteo Foundation, University of Pavia, 27100 Pavia, Italy

**Keywords:** herpesviruses, human cytomegalovirus (HCMV), NK cells, antibodies, non-neutralizing antibodies, antibody-dependent cell cytotoxicity (ADCC)

## Abstract

In the setting of infectious diseases, antibodies show different functions beyond neutralizing activity. In this study, we investigated the activation of NK cells in vitro in the presence of human cytomegalovirus (HCMV)-specific antibodies and their potential role in the control of HCMV infection through antibody-dependent cell cytotoxicity (ADCC). Retinal pigmented epithelial cells (ARPE-19) infected with the HCMV strain VR1814 were co-cultured with cytokine-activated peripheral blood mononuclear cells (PBMCs) in the presence of sera collected from 23 HCMV-seropositive and 9 HCMV-seronegative donors. Moreover, 13 pregnant women sampled 3 and 6 months after HCMV primary infection and 13 pregnant women with pre-conception immunity were tested and compared. We determined the percentage of activated NK cells via the analysis of CD107a expression as a marker of degranulation. Significantly higher levels of NK-cell activation were observed using 1/100 and 1/10 dilutions of sera from HCMV-seropositive individuals, and when cells were infected for 96 and 120 h, suggesting that NK cells are activated by antibodies directed against late antigens. In the absence of serum NK cells, activation was negligible. In seropositive subjects, the median percentages of CD107a-positive NK cells in the presence of autologous serum and pooled HCMV-positive serum were similar (14.03% [range 0.00–33.56] and 12.42% [range 1.01–46.00], respectively), while NK-cell activation was negligible using an HCMV-negative serum pool. In HCMV-seronegative subjects, the median percentage of activated NK cells was 0.90% [range 0.00–3.92] with autologous serum and 2.07% [0.00–5.76] in the presence of the HCMV-negative serum pool, while it was 8.97% [0.00–26.49] with the pool of HCMV-positive sera. NK-cell activation using hyperimmune globulin is comparable to what is obtained using autologous serum. Sera from subjects at 3 and 6 months post primary infection showed a lower capacity of NK-cell activation than sera from subjects with past infection (*p* < 0.001). NK activation against HCMV-infected epithelial cells is dependent on the presence of HCMV-specific antibodies. This serum activity increases with time after the onset of HCMV infection. The protective role of NK-cell activation by HCMV-specific serum antibodies should be verified in clinical settings.

## 1. Introduction

Human cytomegalovirus (HCMV) is the most common congenital infection, affecting 0.5–2% of live births. It can be transmitted after a primary maternal infection, as well as after a non-primary maternal infection, and it accounts for up to 10% of all cases of cerebral palsy and 8–21% of all congenital sensorineural hearing loss at birth [1]. Transmission after primary infection occurs in about 32% of cases; this percentage drops after a non-primary infection, but maternal immunity does not completely prevent vertical transmission. In fact, studies have demonstrated that the birth prevalence of congenital HCMV infection (cCMV) increases with maternal HCMV seroprevalence [2]. Moreover, infection can cause life-threatening HCMV disease in immunocompromised patients, like transplanted patients or people with AIDS. At the moment, an HCMV vaccine is not available [3]. The main immunological endpoints in vaccine development involve neutralizing antibodies against HCMV envelope glycoproteins (e.g., glycoprotein B-gB- and gHgLpUL128-130-131 pentamer complex). In addition, T-cell responses, in particular HCMV-specific CD4+ T cells, have been investigated in vaccine design. However, vaccines tested in clinical trials up to now have achieved only moderate efficacy [4].

There are different studies that show how maternal neutralizing antibody titers do not correlate with a reduced risk of cCMV infection [5,6]. Moreover, two different clinical trials showed that the use of HCMV hyperimmune globulin from HCMV-seropositive donors did not prevent vertical transmission following a primary infection [7,8]. Conversely, an observational study adopting a different posology of hyperimmune globulin reported promising results [9]; thus, an improved understanding of the maternal antibody responses that protect against vertical transmission is needed to guide the development of vaccines [10] or immunotherapies.

Antibodies have different functions beyond their neutralizing activity, and recently, these non-neutralizing functions, such as antibody-dependent cellular phagocytosis (ADCP) and antibody-dependent cellular cytotoxicity (ADCC), have been the target of different studies aiming to understand their potential protective role against HCMV infection [11,12] and against HCMV vertical transmission [13]. The present study will focus on a methodology to test the in vitro capacity of antibodies to activate natural killer (NK) cells, and compare NK activity against HCMV infection in pregnant women with primary HCMV infection and pregnant women with pre-conception HCMV immunity.

## 2. Materials and Methods

### 2.1. Study Population

We analyzed serum and peripheral blood mononuclear cell (PBMC) samples from 29 HCMV-seropositive donors and 9 HCMV-seronegative donors to set up the assay. In addition, the sera from 13 pregnant women with primary HCMV infection at 3 and 6 months post infection, and 13 pregnant women with pre-conception immunity who did not transmit the infection were analyzed for their capacity of inducing ADCC and compared. HCMV-specific IgG was determined by the LIAISON CMV IgG II assay (DiaSorin, Saluggia, VC, Italy). This retrospective study was performed according to guidelines and was approved by the Ethics Committee and Fondazione IRCCS Policlinico San Matteo Institutional Review Board (Procedure n° P-20180075214, P-20170011101 and P-20160034352). All patients signed an informed consent form.

### 2.2. Isolation of PBMCs and Stimulation of NK Cells

The PBMCs obtained were separated by the Lymphoprep (Axis-Shield PoC, Oslo, Norway) density gradient centrifugation of heparinized blood and stored in liquid nitrogen. The day before the experiment, thawed PBMCs were cultured overnight at 37 °C and 5% CO_2_ in RPMI 1640 (Euroclone, Pero, Italy) + 10% heat-inactivated fetal bovine serum (FBS, Euroclone) supplemented with IL-2 and IL-12 (at 100 U/mL and 10 ng/mL, respectively, Immunotools, Friesoythe, Germany) in a 24-well plate at a concentration of 10^6^ PBMCs in 1 mL of medium per well. For all the experiments (except for the set-up part) PBMCs from a single HCMV-seropositive donor were used.

### 2.3. HCMV Strain

An endothelial and epithelial cell-adapted HCMV clinical isolate (VR1814) was used to perform the experiments. The virus was extensively propagated on human umbilical vein endothelial cells for a variable number of passages, until a cell-free virus stock with a sufficient infectious titer was obtained.

### 2.4. Preparation and Infection of Target Cells

In a flat-bottomed 96-well plate, 30,000 ARPE-19 cells/well in 100 uL of DMEM F12 (Euroclone) + 10% heat-inactivated FBS were seeded. After 24 h, the cells were infected with VR1814 at an MOI of 10 in 50 uL DMEM F12 + 2% FBS. The plate was centrifuged for 30′ at 600 g to promote virus–cell contact, and then incubated at 37 °C and 5% CO_2_. After 2 h, the virus-containing medium was removed and substituted with 100 uL of DMEM F12 + 2% heat-inactivated FBS/well. The plate was incubated at 37 °C and 5% CO_2_ until the day of the experiment. Different hours of infection were tested to set up the experiment (24, 48, 96, and 120 h). ARPE-19 cell infection was verified by staining using a monoclonal antibody directed against p72 (IE1), an immediate early protein.

### 2.5. NK Cell CD107a Degranulation Assay

PBMCs were resuspended in RPMI 1640 w/o FBS and seeded 400,000/well over the infected ARPE-19 cells, after the removal of their medium. The serum of patients was added to the co-culture of PBMC-infected ARPE-19 at different dilutions after complement heat-inactivation. An HCMV-seropositive serum pool and an HCMV-seronegative serum pool were added in each test as controls. Moreover, the hyperimmune globulin (HIG) preparation Cytotect 50 U/mL (100 mg of protein of plasma origin per milliliter, resulting in ≥95% IgG, Biotest, Dreieich, Germany) was tested at a concentration of 100 µg/mL. To verify NK activation, K562 cells were used and co-cultured with the PBMCs of 3 controls subject. The BV510-conjugated CD107a mAb (clone H4A3, BD Biosciences, Franklyn Lakes, NJ, USA) was added in each well (5 µL/well), together with Brefeldin A and Monensin (2 mg/mL and 5 mg/mL, respectively, Merck, Darmstadt, Germany).

Following incubation for 4 h at 37 °C and 5% CO_2_, PBMCs were tested for degranulation via the flow cytometry measurement of CD107a expression. PBMCs were collected and transferred in a new, round-bottomed 96-well plate and washed with PBS 2 mM EDTA. They were then stained, for 30 min at 4 °C, with PeCy7-conjugated mAb CD56 (clone B159, BD Biosciences), PercPy5.5-conjugated mAb CD3 (clone UCHT1, BD Biosciences), Pacific Blue-conjugated mAb CD57 (clone NC1, Beckman Coulter, Brea, CA, USA), and APC-conjugated mAb anti-NKG2C (CD159c, clone # 134591, Bio-techne, Minneapolis, MN, USA) in PBS + 5% FBS. Cells were then washed with PBS + 5% FBS and fixed in PBS 1% paraformaldehyde.

Using an FACS CantoII flow cytometer and FACSDiva™ software (BD Biosciences), the percentage of CD107a-positive CD3^−^CD56^+^ or CD3^−^CD56^+^CD57^+^ cells was determined. CD107a is a marker of cell degranulation and its expression on NK cells is a marker of NK-cell activation.

### 2.6. Statistical Analysis

Data on NK-cell activation are expressed as the median with a range. The non-parametric Friedman test and the Mann–Whitney U were used to compare the percentage of CD107a-positive cells in different test conditions and the levels of HCMV-specific IgG. The Spearman r test was used to analyze the correlation between the percentage of CD107a-positive cells and the levels of HCMV-specific IgG. Analyses were performed with Prism 8.3.0 Software (Graph Pad Software, San Diego, CA, USA).

## 3. Results

### 3.1. Optimization of Serum Dilution

**Figure 1:**
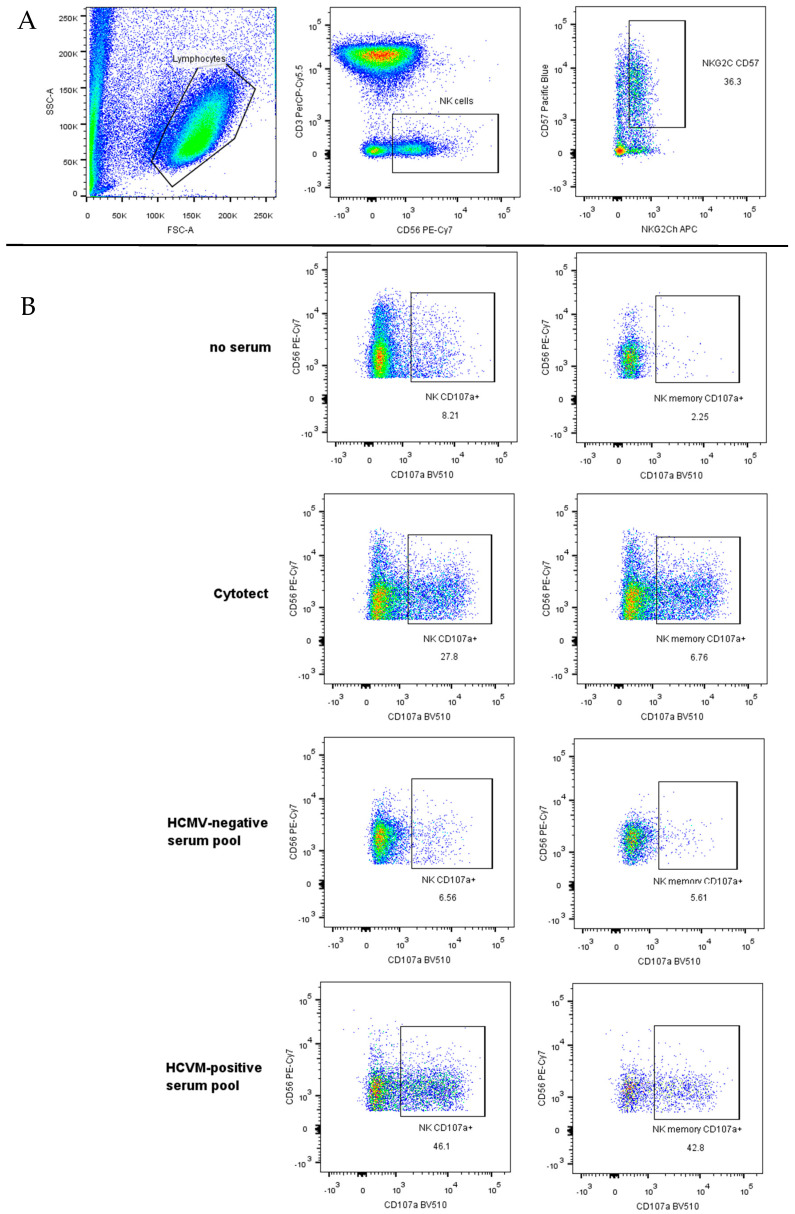
Percentage and representative plots of NK cells (CD3^−^CD56^+^) on the left side, and memory NK cells (NKG2c^high^CD57^+^) on the right (**A**). Representative plots of CD107a expression after co-culture with VR1814-infected ARPE-19 cells without serum, with Cytotect, and in the presence of HCMV-negative serum pool and HCMV-positive serum pool (**B**).

PBMCs obtained from six seropositive donors were used for the optimization of the serum dilution. The serum used for the set-up was a pool of serum of HCMV-seropositive donors. Four different dilutions were tested: 1/10, 1/100, 1/1000, and 1/10,000. The sera were diluted in the same medium used for the PBMCs: RPMI 1640 w/o FBS. ARPE-19 target cells were used 120 h post infection. The percentage of CD107 expression was calculated, as marker of degranulation, on NK (CD3^−^CD56^+^) cells and memory (NKG2c^high^CD57^+^) NK cells (Figure 1A,B).

**Figure 2:**
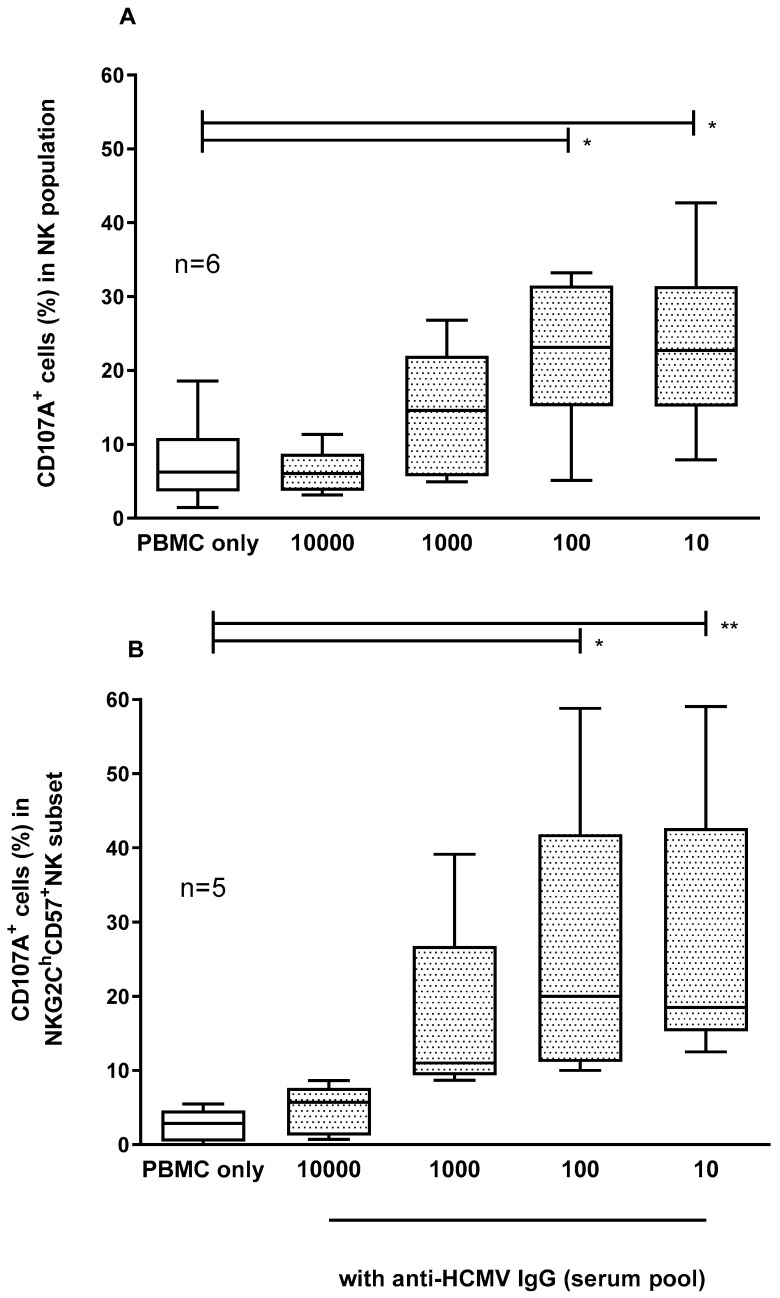
Percentages of CD107a expression in NK cells (**A**) and memory NK cells (**B**) of 6 HCMV-seropositive subjects with anti-HCMV IgG after 4 h of co-culture with VR1814-infected ARPE-19 cells. Median, 25–75 percentile, and min to max are represented for each condition. * *p* < 0.05; ** *p* < 0.01.

Significantly higher levels of NK-cell and memory NK-cell activation were observed using 1/100 and 1/10 serum dilutions compared with the PBMCs alone (medians for CD107a-positive cells: 23% for both dilutions for the NK cells, and 20% and 19%, respectively, for the memory NK cells, Figure 2A,B). Therefore, the 1/100 dilution was chosen for the subsequent experiments.

### 3.2. Time Post Infection and NK Activation

**Figure 3:**
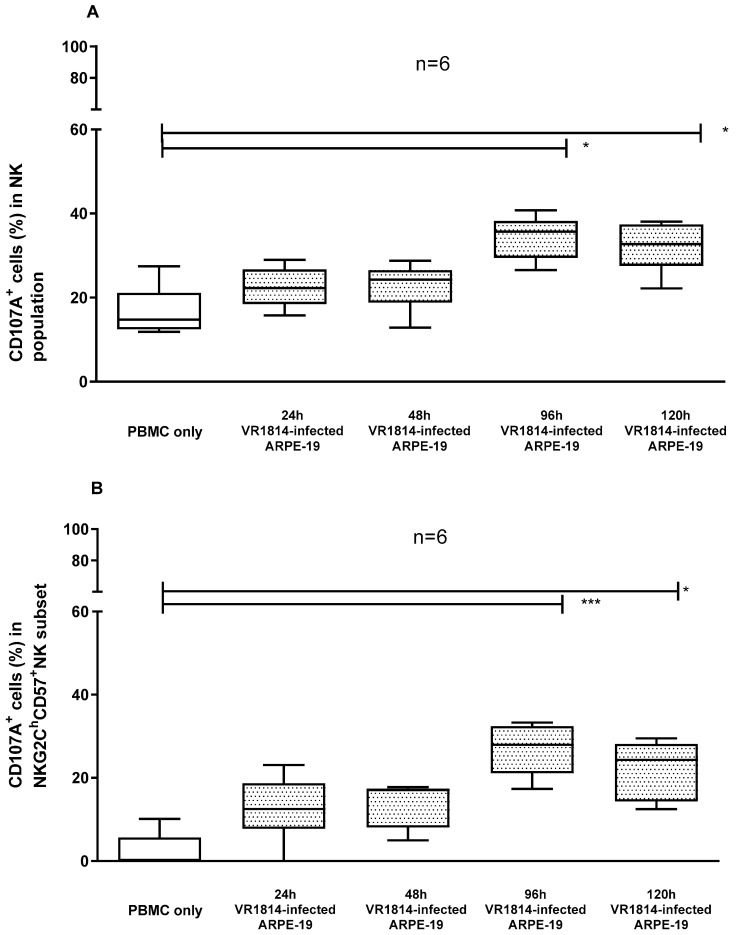
Percentages of CD107a expression in NK cells (**A**) and memory NK cells (**B**) of six HCMV-seropositive subjects after 4 h of co-culture with VR1814-infected ARPE-19 cells at different hours of infection. Median, 25–75 percentile, and min to max are represented for each condition. In the box of 48 h VR1814-infected ARPE-19 in panel B, the 75th percentile is overlapping with the median. * *p* < 0.05; *** *p* < 0.001.

Different hours of infection of ARPE-19 cells were tested (Figure 3). The NK cells from a single donor were used together with the sera of six subjects at a dilution of 1/100. A similar percentage of response was obtained with 24 and 48 h of infection for both the NK cells (Figure 3A median: 22% and 24%, respectively) and the memory NK cells (Figure 3B median: 13% and 17%, respectively). Significantly higher levels of CD107a expression were seen when cells were infected for 96 and 120 h, compared with the PBMCs alone (Figure 3A median: 36% and 33%, respectively); this difference remained significant after 96 and 120 h of infection for the memory NK cells (Figure 3B median: 28% and 24%, respectively). In the subsequent experiments, ARPE-19 cells 120 h post infection were chosen as target cells for experimental practicality.

### 3.3. NK-Cell Activation in HCMV-Seropositive and -Seronegative Subjects

**Figure 4:**
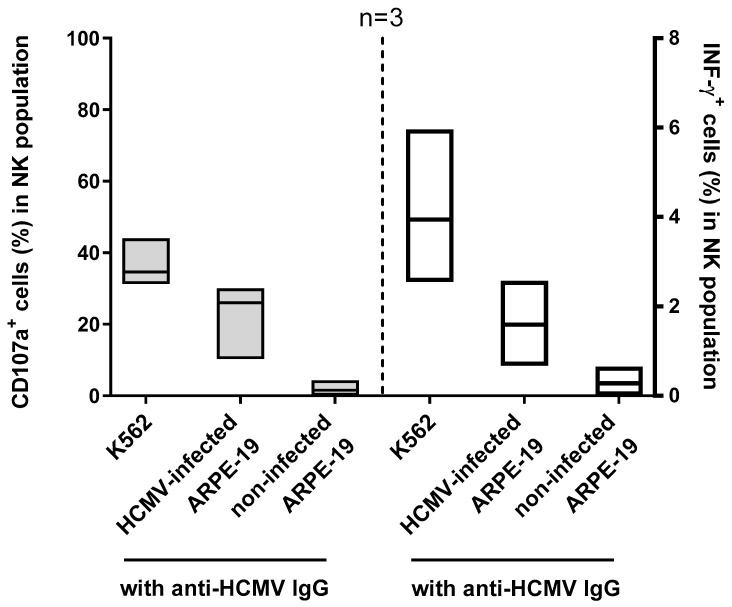
Percentages of NK cells expressing CD107a or INF-γ after co-culture with K562 cells, VR1814-infected cells, or non-infected ARPE-19 cells in the presence of anti-HCMV IgG (HCMV-seropositive serum). Median and min to max are represented for each condition.

After the set-up of the experiment, we tested three HCMV-seropositive subjects to verify that serum from HCMV-seropositive subjects activates NK cells only in the presence of HCMV-infected cells. Moreover, INF-γ production was also analyzed in addition to CD107a expression. K562 cells were used to control NK-cell activation. The results were calculated as the difference between CD107a^+^ cells and INF-γ production in the presence of serum and the percentage of CD107a^+^ cells and INF-γ production with PBMCs alone. NK cells were activated by HCMV-seropositive serum when co-cultured with HCMV-infected ARPE-19 cells, but not with non-infected cells. Yet, as expected, the K562 cells were capable of inducing NK-cell activation. We observed a similar pattern in the percentage of INF-γ-producing NK cells in the same condition of co-cultivation; however, the percentage of INF-γ-positive cells was ten times lower than that of CD107a-positive cells, with both using HCMV-infected cells plus serum or K562 cells as stimuli (Figure 4).

**Figure 5:**
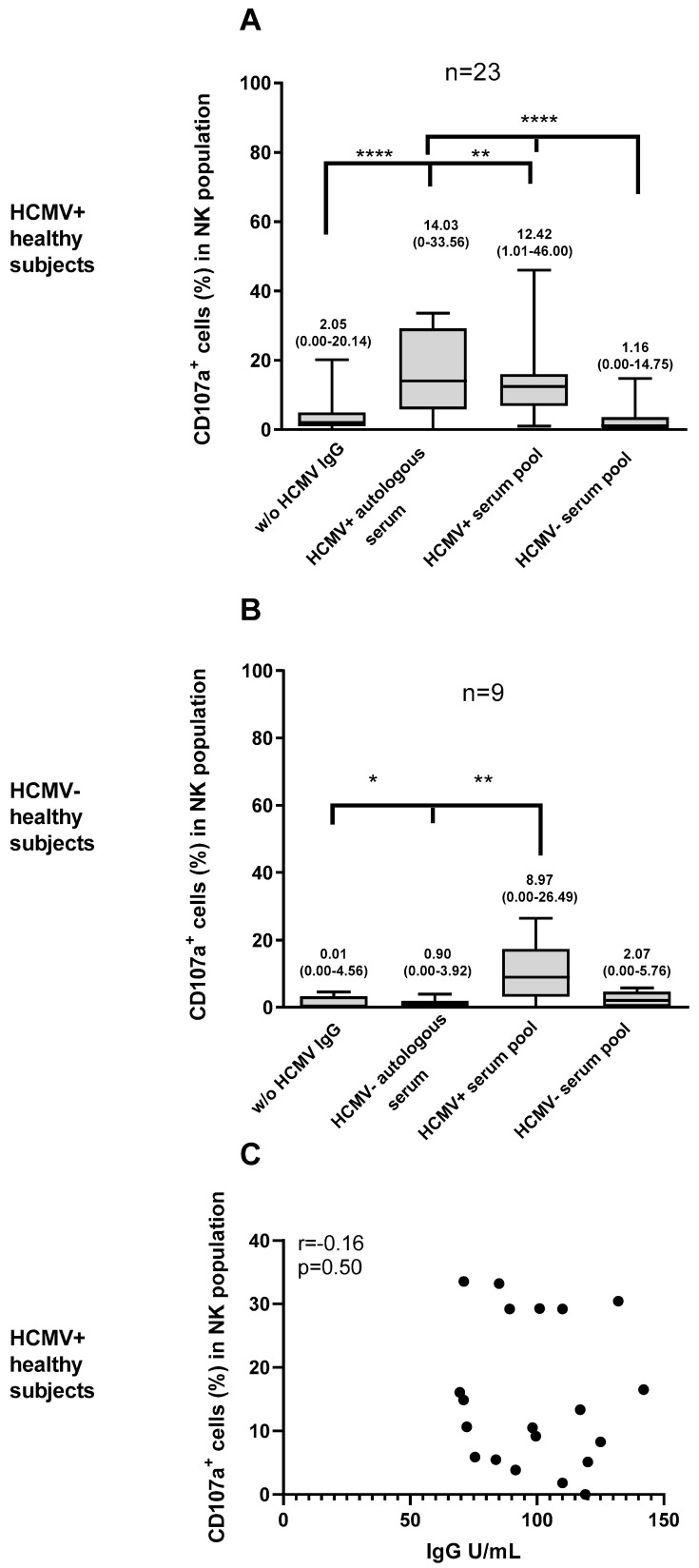
Percentages of CD107a expression in NK cells of 23 HCMV-seropositive subjects (**A**) and 9 HCMV-seronegative subjects (**B**) after co-culture with VR1814-infected ARPE-19 cells using autologous serum, HCMV-seropositive serum pool, and HCMV-seronegative serum pool. Median, 25–75 percentile, and min to max are represented for each condition. Correlation between CD107a expression on NK cells and levels of HCMV-specific IgG (U/mL) in HCMV-seropositive subjects (**C**). *, *p* < 0.05; ** *p* < 0.01; **** *p* < 0.0001.

Finally, 23 HCMV-seropositive and 9 HCMV-seronegative subjects were tested, using both the autologous serum and the HCMV-seropositive and HCMV-seronegative serum pools (Figure 5). The results were calculated as the difference between CD107a^+^ cells in the presence of serum and the percentage of CD107a^+^ cells with PBMCs alone.

In the HCMV-seropositive subjects, the median percentages of CD107a^+^ NK cells in the presence of autologous serum and the HCMV-seropositive serum pool were similar (median: 14% [0–34%] and 12% [1–46%], respectively), while NK-cell activation with the HCMV-seronegative serum pool was negligible (median: 1% [0–15%]). A statistically significant difference was observed when comparing the percentage of NK cells activated by the autologous serum and the response obtained with the HCMV-seronegative serum pool or no serum (*p* < 0.001). This difference persisted when analyzing the response with the HCMV-seropositive serum pool (Figure 5A *p* < 0.001 and *p* = 0.002).

In HCMV-seronegative subjects, the median percentage of NK-cell activation with autologous serum was 1% [0–4%]; with the HCMV-seronegative serum pool, the median was 2% [1–6%], and in the presence of the HCMV-seropositive serum pool, the median was 9% [0–26%]. A statistically significant difference was observed when comparing the percentage of NK cells activated by the HCMV-seropositive serum pool and the response obtained with the autologous serum pool and no serum (Figure 5B *p* = 0.004 and *p* = 0.02).

We verified if there was an association between the production of CD107a by NK cells and the level of HCMV-specific IgG, but no correlation was found. The percentage of activated NK cells is therefore independent of the level of HCMV-specific IgG present in the serum (Figure 5C).

**Figure 6:**
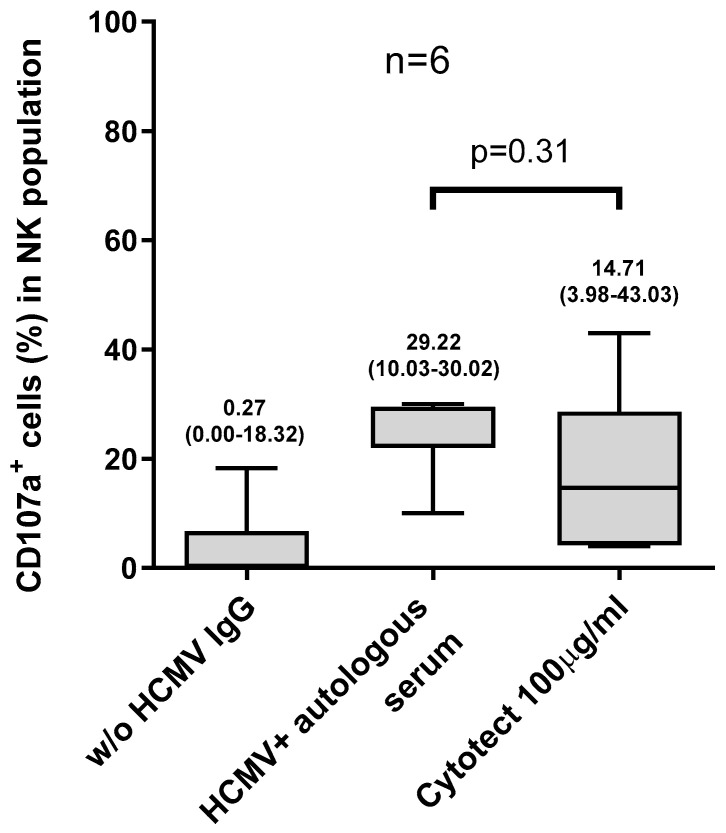
Percentages of CD107a expression in NK cells of 6 HCMV-seropositive subjects after co-culture with VR1814-infected ARPE-19 cells using autologous serum 1/100, Cytotect 100 µg/mL, and without serum. Median, 25–75 percentile, and min to max are represented for each condition.

In six HCMV-seropositive subjects, NK-cell activation was assessed using an HIG preparation and confronted with the NK-cell activation obtained with the autologous serum. HIG was used at a final concentration of 100 µg/mL of IgG, which is similar to that present in human serum diluted 1/100. NK-cell activation by HIG (median: 15% [4–43%]) was similar to that observed with human serum (median: 29% [10–30%]). NK-cell activation without serum was negligible (median: 0% [0–18%]) (Figure 6, *p* = 0.31).

### 3.4. NK-Cell Activation in Pregnant Women with HCMV Primary or Pre-Conception Infection

**Figure 7:**
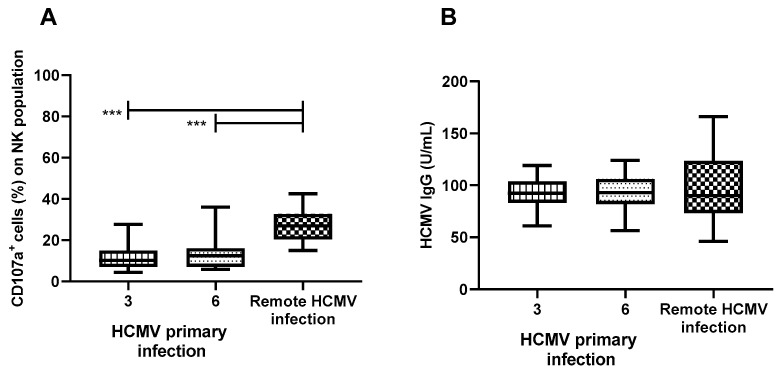
Percentages of CD107a expression in NK cells of HCMV-seropositive donors after co-culture with VR1814-infected ARPE-19 cells using sera of 13 pregnant women with primary HCMV infection (3 and 6 months post infection) and 13 pregnant women with HCMV pre-conception immunity (**A**). Levels of anti-HCMV IgG (U/mL) in the serum of 13 pregnant women with primary-HCMV infection (3 and 6 months post infection) and 13 pregnant women with HCMV pre-conception immunity (**B**). Median, 25–75 percentile, and min to max are represented for each condition. *** *p* < 0.001.

We tested the sera of 13 pregnant women at 3 and 6 months post primary infection and 13 pregnant women with HCMV pre-conception immunity to verify whether the ability of serum antibodies to activate NK cells is different in the two conditions of HCMV infection. PBMCs of a single HCMV-seropositive donor were used as effector cells. The results were calculated as the difference between CD107a^+^ cells in the presence of serum and the percentage of CD107a^+^ cells with PBMCs alone. Serum from women with HCMV primary infection showed a low capacity of NK-cell activation (median: 10% [4–28%] at 3 months post infection and 12% [6–36%] at 6 months) compared to serum from women with past infection (median: 26% [13–45%]; *p* < 0.001 for both time points; Figure 7A).

We compared the levels of HCMV-specific IgG between the HCMV primary infection at 3 and 6 months after the onset of infection and the HCMV pre-conception infection. No significant difference was observed (Figure 7B). The difference in NK-cell activation was not due to the level of HCMV-specific IgG.

## 4. Discussion

In this study, we developed an assay to analyze the activation of NK cells by HCMV-infected epithelial cells. As a marker of NK-cell activation, we analyzed the expression of CD107a on the cell surfaces. Although we did not measure actual cell killing, CD107a expression is associated with degranulation and is widely considered a surrogate of cytotoxic activity. The major results of our study indicate that NK-cell activation against HCMV-infected cells was dependent on the presence of HCMV-specific antibodies (Figure 6). In fact, HCMV-infected cells were only able to activate NK cells in the presence of human serum containing HCMV-specific antibodies (either from an autologous or heterologous donor). Conversely, NK cells co-cultured with HCMV-infected cells without human serum showed no sign of degranulation (i.e., CD107a expression). Similarly, the activation of NK cells was almost negligible when serum from an HCMV-seronegative donor (therefore not containing HCMV-specific antibodies, Figure 5) was used. On the other hand, HCMV-specific antibodies do not activate NK cells co-cultured with mock-infected cells (Figure 4).

The lack of NK-cell activation by HCMV-infected cells alone could be due to the array of gene products and microRNA that express HCMV and are capable of suppressing NK-cell recognition [14]. For example, the role of UL18 glycoprotein in HCMV’s evasion of NK cells by its interaction with LIR-1 (inhibitory leukocyte Ig-like receptor) has been described [15].

In our experimental conditions, the role of the so-called “memory-like” NK cells (expressing NKG2C and CD57) did not seem predominant in ADCC (Figure 2 and Figure 3). It has been shown that the expansion of NKG2C^+^ NK cells is associated with HCMV infection, and that they preferentially acquire CD57 [16,17,18]. These cells are very efficient at mediating ADCC and have been associated with protection from HCMV disease in transplant patients [19]. Using 24 h HCMV-infected (TB40/E strain) macrophages as target cells, Wu and colleagues [17] observed a lower activation of NKG2C-positive than NKG2C-negative cells in the absence of antibodies, whereas the presence of anti-HCMV antibodies was required to activate NKG2C-positive cells. In our model, we observed a negligible activation of both total and NKG2C-positive NK cells in the absence of anti-HCMV antibodies, and similar percentages of activated NK cells among total or “memory-like” NKG2C-positive NK cells when anti-HCMV antibodies were present. Furthermore, we observed that NK cells of HCMV-seronegative subjects are activated in a comparable way to that of HCMV-seropositive subjects when in presence of anti-HCMV antibodies. Therefore, NK cells appear prone to activation by anti-HCMV antibodies, regardless of their previous encounters with HCMV or NKG2C expression.

Different times of HCMV infection were tested during the setting of the experiment (Figure 3). The best results were obtained in the late stage of infection, after 96 and 120 h. This suggests that NK cells are activated by antibodies directed against late antigens, at least when tested against ARPE cells. This is in contrast with what was found in another study [20], where the authors demonstrated that the viral antigens responsible for activating ADCC, surprisingly, were not structural glycoproteins, but glycoprotein expressed during the early phase of infection, meaning that anti-HCMV antibodies could activate NK cells early after HCMV infection, prior to the production of new virions. These contrasting results may be partly due to the different target cells (ARPE vs. fibroblasts) and HCMV virus strains (VR1814 vs. Merlin) used in the two different studies.

Comparing the ability to initiate ADCC between the sera of pregnant women with HCMV primary infection at 3 and 6 months post infection and women with pre-conception infection, it emerges that with the progression of the infection this ability increases significantly (Figure 7, *p* < 0.001). We also analyzed the levels of HCMV-specific IgG between the three groups, but no difference emerged. The different levels of NK-cell activation are likely due to the quality of IgG antibodies (e.g., binding capacity to Fc receptors) rather than to their quantity. Permar and colleagues confronted the ability of ADCC in women who did or did not transmit HCMV to the fetus during pregnancy, finding that the presence of ADCC-activating antibodies correlates with a decreased risk of congenital transmission [13]. As stated by the authors, in their study, it was not possible to determine the timing of maternal HCMV exposure. Considering the significant difference in ADCC ability that we saw between HCMV primary and pre-conception infection, it is plausible to assume that the different ADCC activity observed in the two groups in that study was associated with different HCMV infection conditions, comparable to primary (when transmission risk is high) and preconception HCMV infections (when transmission risk is much lower).

In our study, we tested the ability to initiate ADCC only in response to HCMV-infected epithelial cells. As a next step, the response of NK cells to HCMV infection in other target cells, e.g., fibroblast and endothelial cells, should be investigated, along with actual cell killing. The experiments on primary and remote HCMV infections were not performed using autologous PBMCs, which were not available, but using the cells of one HCMV-seropositive donor. This donor was chosen due to the fact that they had an FcγRIII-158 V polymorphism, which confers a high binding affinity against antibodies [21]. Therefore, we could evaluate the different antibody capabilities of activating NK cells in the two conditions of primary and chronic infection. In future studies, the use of autologous PBMCs could also provide information on the individual variability in NK-cell capability to be activated by antibodies and infected cells. The major limitation of our study is the fact that the target antigens responsible for the activation of ADCC were not explored. The identification of the antigen specificity of the NK-activating antibodies would provide important information on the potential utility of specific monoclonal antibodies in therapy settings, beyond sole neutralizing activity [22,23]. This is very important, since neutralizing antibodies does not seem to be protective against HCMV infection or HCMV transmission [5], probably due the fact that it is a cell-associated virus that has the ability to spread directly, cell to cell [24,25]. ADCC, and therefore non-neutralizing antibodies, could be more effective in the elimination of infected cells and in blocking the spread of the virus. Moreover, from our study, it has emerged that the use of HIG gives results of NK-cell activation comparable to that obtained with autologous serum; therefore, the use of HIG in clinical settings may also exploit NK-cell activation.

On the other hand, an advantage of our experimental setting is the use of HCMV-infected cells as a target of ADCC. This setting is much more physiological, in terms of antigen expression on the cell surface, than the use of single antigen-transfected cells or antigen-coated beads, and provides a better in vitro surrogate to gain information on how the non-neutralizing antibodies response against HCMV infection works in vivo.

More studies are needed to better understand the role of this type of antibodies, and the protective role of NK-cell activation by HCMV-specific serum antibodies should be verified in clinical settings.

In the future, the role of ADCC in protecting against HCMV vertical transmission should be verified, as well as its role in the control of HCMV infection in immunocompromised transplanted patients. If an essential role of ADCC in virus control is demonstrated, immunotherapies with antibodies capable of inducing ADCC could be taken into account.

It is, however, known that individuals lacking functional NK cells suffer severe, recurrent infections of HCMV [26,27], highlighting the critical role of NK cells in infection control. Therefore, an efficacious vaccine to prevent HCMV infection should potentially aim to elicit NK-cell antiviral responses, as well as conventional T and B cells.

## Data Availability

The data presented in this study are available on request from the corresponding author.
